# Internal Auditory Canal Dural Enhancement in Granulomatosis with Polyangeitis Disease

**DOI:** 10.1155/2018/2171434

**Published:** 2018-05-21

**Authors:** Juan Carlos Izquierdo Velásquez, Luis Felipe Romero Moreno

**Affiliations:** ^1^Department of Otology and Neurotology, Colombian National University Hospital, Bogotá, Colombia; ^2^Department of Otolaryngology Head and Neck Surgery, National University of Colombia, Bogotá, Colombia

## Abstract

Diffuse dural enhancement of the internal auditory canal in T1-weighted gadolinium-enhanced magnetic resonance imaging could be a helpful and early clinical sign in a very aggressive limited granulomatosis with polyangeitis disease, called previously Wegener Disease* (WD)*.

## 1. Introduction

Granulomatosis with polyangeitis disease is a systemic entity mediated by autoimmune necrotizing inflammation that has the ability to affect every microsystem of the human body that was related with antineutrophil cytoplasmic antibody* (ANCAS)* activity [1]. Central nervous system* (CNS)* can be compromised in almost 50% of cases; however, most symptoms occur in very advanced disease states, so finding the neurological involvement like the first symptom of the disease tends to be very unusual [2]. Otologic symptoms such as sudden sensorineural hearing loss and facial paralysis as initial manifestations in patient with* GPA* are extremely rare. The prevalence can vary from 4 to 12% depending on series [2]. Other possible nasosinusal symptoms include septal perforation (4%), chronic dacryocystitis (4%), and tracheal stenosis (1%) [1–3].

The systemic type represents the majority of cases of* GPA*, typically affecting the high and low respiratory tract, and it has been associated with focal segmental glomerulonephritis. The localized* GPA* is not as common as the systemic* GPA* and affects just a single or a few organs without renal compromise [4]. When presented ENT is compromised, hearing loss can develop rapidly or gradually over several days or weeks. Although the exact cause still remains unknown, some theories link it to granulomatous compression of the cochlear nerve, deposits of immunocomplexes in the cochlea, and vasculitis of the* vasa nervorum *and cochlear vessels [3, 5].

We report the radiological and histopathology findings of a very aggressive limited* GPA*, after approval of the ethics committee of the Central Police Hospital, Bogota, Colombia.

## 2. Clinical Case

A 47-year-old male patient presented with a two-month history of rapid progressive bilateral hearing loss, unilateral facial paralysis, and severe headaches.

**Figure 1:**
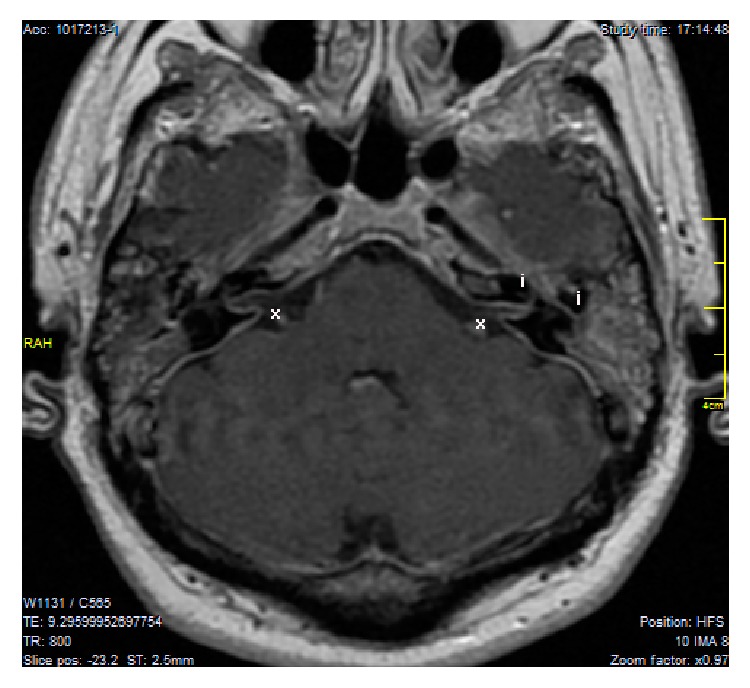
*T1-weighted Gd-MRI, axial image*. x: symmetrical lineal diffuse thickening and enhancement of the dural layer of the IAC. i: left labyrinthine and tympanic facial nerve enhancement.

The physical exam showed bilateral serous otitis media, House & Brackmann VI/VI grade left peripheral facial paralysis, and bilateral severe to profound sensorineural hearing loss. No other additional systemic or neurological deficiencies were found. We found in the T1-weighted, gadolinium-enhanced magnetic resonance imaging* (Gd-MRI)* diffuse dural and symmetrical enhancement of the dural layer of the posterior and middle fossae including the convexity and surprisingly the dural layer of the internal auditory canal* (IAC)* and the left labyrinthine and tympanic facial nerve (Figure 1).

**Figure 2:**
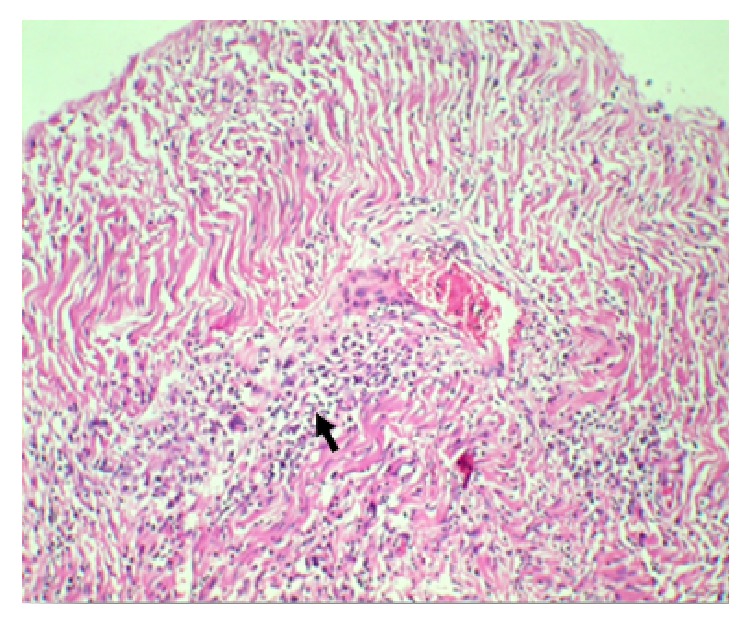
*Dura (haematoxylin and Eosin)*.* Arrow*: multinucleated giant cells associated with the small caliber vessel* wall. Arrow*: severe mononuclear infiltrate.

Suddenly after a complete radiological and serological study battery the patient developed a severe respiratory insufficiency and died. The diagnosis of GPA was based on the clinical and serologic criteria of the American College of Rheumatology [6] and confirmed after the autopsy and histopathology results. The histopathological study evidences severe mononuclear infiltrate of the dura and multinucleated giant cells associated with the wall small caliber vessel. The last finding could be proposed to be considered as pathognomonic sign of* GPA* (Figure 2).

## 3. Discussion

Drachman et al. [7] found three different pathological patterns of compromise in CNS in patients with* GPA* disease. Head and neck region is the most frequent anatomical location for manifestations of* GPA*. There exist two main mechanisms through the disease involving the brain. One of those is hematogenous spread, in which disseminated vasculitis involving both small arteries and veins occurs to a greater or lesser degree as the disease progresses. A localised form of* GPA* limited primarily to the upper and lower respiratory tracts has been described [3, 8]. It is important to keep in mind the differential diagnosis of dilated cardiomyopathy, especially in the existence of pulmonary and renal pathologies [4].

The second mechanism of dissemination is through paranasal disease. Up to 30% of patients only present nasal symptoms, with the most common being nasal obstruction and rhinorrhea. Frontal sinus invasion and skull base compromised at the sphenoid sinus can represent a risk factor for brain involvement [9]. The granulomatous pathological pattern causes meningeal thickening like in other kinds of entities such as neurosarcoid, neurosyphilis, pachymeningitis, and lymphoma [5, 7].

Murphy et al. [10] divided the image findings into two classes, the T1-weighted* (Gd-MRI)* dural findings in focal thickening and enhancement associated with parameningeal disease or diffuse thickening and enhancement without association with parameningeal disease. Our case, which corresponded to a limited type of GPA, was presented with nonspecific symptoms of severe headache, facial and auditory nerve involvement, and focal dural thickening in T1 (Gd-MRI) with associated enhancement.

The diagnosis in this patient was certainly a challenge. Unfortunately the medical exercise performed on him and the rapid progression of the disease were not enough for having a timely diagnosis. A few days after income, the patient presented a massive pulmonary hemorrhage leading to death at the intensive care unit.

## 4. Conclusion

We believe that the T1-weighted* Gd-MRI* dural* IAC* enhancement could be a very helpful sign in limited* GPA*, especially when the clinical presentation including unique otological symptoms.

## References

[1] Vega Braga F. L., Machado de Carvalho G., Caixeta Guimarães A., Scaramussa L., Jordão Gusmão R. (2013). Otolaryngological Manifestations of Wegener's Disease. *Acta Otorrinolaringologica (English Edition)*.

[2] Haas J.-P., Metzler M., Ruder H., Waldherr R., Böswald M., Rupprecht T. (2002). An unusual manifestation of Wegener's granulomatosis in a 4-year-old girl. *Pediatric Neurology*.

[3] Sharma A., Deshmukh S., Dabholkar J. (2013). ENT manifestations of Wegeners granulomatosis. *Otolaryngologia Polska*.

[4] Safak O., Gursul E., Polat M. (2016). Wegener's granulomatosis with cardiac involvement. *Gynecologic Oncology Reports*.

[5] Dutra L. A., de Souza A. W. S., Grinberg-Dias G., Barsottini O. G. P., Appenzeller S. (2017). Central nervous system vasculitis in adults: An update. *Autoimmunity Reviews*.

[6] Lavitt R. Y., Fauci A. S., Bloch D. A., The American College of Rheumatology (1990). Criteria for the classification of Wegener's Granulomatosis. *Arthritis & Rheumatism*.

[7] Drachman D. A. (1963). Neurological Complications of Wegener's Granulomatosis. *JAMA Neurology*.

[8] Nagashima T., Maguchi S., Terayama Y. (2000). P-ANCA-positive Wegener's granulomatosis presenting with hypertrophic pachymeningitis and multiple cranial neuropathies: Case report and review of literature. *Neuropathology*.

[9] Ferri E., Armato E., Capuzzo P., Cavaleri S., Ianniello F. (2007). Early diagnosis of Wegener's granulomatosis presenting with bilateral facial paralysis and bilateral serous otitis media. *Auris Nasus Larynx*.

[10] Murphy J. M., Gomez-Anson B., Gillard J. H. (1999). Wegener granulomatosis: MR imaging findings in brain and meninges. *Radiology*.

